# Anti-Inflammatory Effects of a Polyphenol, Catechin-7,4′-*O*-Digallate, from *Woodfordia uniflora* by Regulating NF-κB Signaling Pathway in Mouse Macrophages

**DOI:** 10.3390/pharmaceutics13030408

**Published:** 2021-03-19

**Authors:** Eui Jin Kim, Ji Bin Seo, Jae Sik Yu, Seoyoung Lee, Jae Sung Lim, Jeong Uk Choi, Chang-Min Lee, Luay Rashan, Ki Hyun Kim, Young-Chang Cho

**Affiliations:** 1 Research Institute of Pharmaceutical Sciences, College of Pharmacy, Chonnam National University, Gwangju 61186, Korea; jin2kim6999@daum.net (E.J.K.); wlqls6208@naver.com (J.B.S.); 96_29@naver.com (S.L.); cju0667@jnu.ac.kr (J.U.C.); 2 School of Pharmacy, Sungkyunkwan University, Suwon 16419, Korea; jsyu@bu.edu; 3 Combinatorial Tumor Immunotherapy Medical Research Center, Chonnam National University Medical School, Jeonnam 58128, Korea; dr.jslim7542@gmail.com; 4 Department of Biochemistry, Chonnam National University Medical School, Jeonnam 58128, Korea; 5 Department of Veterinary Medicine, College of Veterinary Medicine and BK21 FOUR Program, Chonnam National University, Gwangju 61186, Korea; cmlee1122@jnu.ac.kr; 6 Research Center, Biodiversity Unit, Dhofar University, Salalah 211, Oman; lrashan@du.edu.om

**Keywords:** *Woodfordia uniflora*, polyphenol, macrophages, inflammation, NF-κB, Arid5a

## Abstract

Inflammation is a defense mechanism that protects the body from infections. However, chronic inflammation causes damage to body tissues. Thus, controlling inflammation and investigating anti-inflammatory mechanisms are keys to preventing and treating inflammatory diseases, such as sepsis and rheumatoid arthritis. In continuation with our work related to the discovery of bioactive natural products, a polyphenol, catechin-7,4′-*O*-digallate (CDG), was isolated from *Woodfordia uniflora*, which has been used as a sedative and remedy for skin infections in the Dhofar region of Oman. Thus far, no study has reported the anti-inflammatory compounds derived from *W. uniflora* and the mechanisms underlying their action. To investigate the effects of CDG on the regulation of inflammation, we measured the reduction in nitric oxide (NO) production following CDG treatment in immortalized mouse Kupffer cells (ImKCs). CDG treatment inhibited NO production through the downregulation of inducible nitric oxide synthase expression in lipopolysaccharide (LPS)-stimulated ImKCs. The anti-inflammatory effects of CDG were mediated via the inhibition of nuclear factor kappa-light-chain-enhancer of activated B cells (NF-κB) activation, an important inflammatory-response-associated signaling pathway. Moreover, CDG treatment has regulated the expression of pro-inflammatory cytokines, such as IL-6 and IL-1β. These results suggested the anti-inflammatory action of CDG in LPS-stimulated ImKCs.

## 1. Introduction

Inflammation is a biological response to tissue damage or infection, and the main inflammatory mediators include immune cells, such as macrophages and neutrophils [1,2]. The purpose of inflammation is to suppress tissue damage and regenerate damaged cells. However, chronic inflammation contributes to inflammatory diseases, such as inflammatory bowel disease, sepsis, and rheumatoid arthritis [3,4,5]. Therefore, it is important to control the inflammation levels for the maintenance of homeostasis in the body. Inflammatory responses occur when immune cells in the body recognize infections and then activate the inflammatory pathways, such as the nuclear factor kappa-light-chain-enhancer of activated B cells (NF-κB) pathway [6], and produce inflammatory mediators, such as pro-inflammatory cytokines, nitric oxide (NO), and cyclooxygenase 2 (COX-2) [7,8].

NO is an important signaling molecule involved in various physiological activities, such as immune action, vascular extension, and cell signaling [9]. It triggers a defensive mechanism in the immune system and mediates the activation of various immune cells. When macrophages are exposed to external stimuli, they secrete NO through the activation of inducible nitric oxide synthase (iNOS) as a defensive response [10]. Recent studies have found that excessive NO production can cause diseases such as Parkinson’s disease and ischemic brain injury [11,12]. Therefore, the regulation of NO production is important for maintaining human health [13,14]. Thus, there is a crucial need to discover novel regulators of NO production; natural products represent potential candidate anti-inflammatory mediators.

In continuation of our work related to the discovery of bioactive products from diverse natural sources [15,16,17,18,19,20], a liquid chromatography/mass spectrometry (LC/MS)-guided chemical analysis of the extract of *Woodfordia uniflora* (*W. uniflora*), native plant species of Oman, was performed to identify anti-inflammatory compounds. *W. uniflora* is a tall slender shrub belonging to the family Lythraceae that is abundant throughout the Dhofar region in Oman; the plant has been used locally as a sedative and remedy for skin infection. In our recent study, the first phytochemical examination of *W. uniflora* was conducted [21]; 19 polyphenols, including three new polyphenols, were successfully isolated from the extract and its fractions. These compounds possess antifungal activity against the human fungal pathogens *Cryptococcus neoformans* and *Candida albicans*; however, no anti-inflammatory compounds derived from *W. uniflora* have been identified thus far, and the mechanisms underlying their activity have not yet been elucidated. In the present study, phytochemical analysis of the extract of *W. uniflora* led to the isolation of catechin-7,4′-*O*-digallate (CDG) by LC/MS-guided chemical analysis, and the anti-inflammatory effects of CDG and the mechanisms underlying its action were investigated. Further, we describe the isolation and structural characterization of CDG and demonstrate its effects on inflammatory mediators, such as NO and pro-inflammatory cytokines, and the inflammatory NF-κB signaling pathway in lipopolysaccharide (LPS)-stimulated immortalized mouse Kupffer cells (ImKCs).

## 2. Materials and Methods

### 2.1. Extraction and Isolation

Finely ground leaves (100 g) were extracted with 90% methanol (MeOH) and stirred at room temperature. The resultant extracts were filtered through Whatman’s Grade 1 filter paper, and the collected filtrates were concentrated by using a rotary evaporator to afford a crude extract (12.4 g). This crude extract was suspended in distilled water (700 mL) and successively partitioned with the solvents ethyl acetate (EtOAc) and n-butanol (*n*-BuOH), thus yielding the EtOAc-soluble (8.3 g) and *n*-BuOH-soluble (1.5 g) fractions. The EtOAc-soluble fraction was further subjected to the Diaion HP-20 column in a gradient solvent system consisting of MeOH and H_2_O (100% H_2_O, 50% MeOH, 100% MeOH) to yield three subfractions W1 (2.0 g), W2 (2.5 g), and W3 (3.5 g). The W3 subfraction (100% MeOH) (3.5 g) was applied to Sephadex LH-20 column chromatography to give four successive fractions (W31‒W34). The W34 subfraction (2.7 g) was then fractionated by reversed-phase (RP)-silica column chromatography with 40% MeOH/H_2_O to afford five subfractions (W341‒W345). The W33 subfraction (352.5 mg) was separated using preparative RP HPLC with MeOH/H_2_O (gradient solvent system of 10–80% MeOH/H_2_O) to yield six subfractions (W331‒W336). Finally, the subfraction W334 (92.3 mg) was purified by the semi-preparative HPLC separation with 45% MeOH/H_2_O to isolated CDG (*t*_R_ 12.8 min, 4.5 mg).

### 2.2. Cell Culture

ImKCs (SCC119) were purchased from Sigma-Aldrich (St. Louis, MO, USA). The ImKCs were cultured in Roswell Park Memorial Institute (RPMI) 1640 medium (Sigma-Aldrich) containing 1% antibiotic-antimycotic (100×) (Thermo Fisher Scientific, Inc., Waltham, MA, USA) and 10% Fetal Bovine Serum (FBS; Thermo Fisher Scientific, Inc.) in an incubator at 37 °C under 5% CO_2_ conditions.

### 2.3. Cell Viability Assay

The ImKCs were seeded in a 96-well plate and pretreated with CDG (5, 10, 20, 40, and 80 μM) for 24 h in a CO_2_ incubator at 37 °C. Cell viability was measured using the EZ-Cytox cell viability assay kit (Daeil Lab., Seoul, Korea); the reagent was diluted (1:20) with DMEM. All the supernatants were transferred to another 96-well plate. The EZ-Cytox reagent was added to each well, followed by incubation in a CO_2_ incubatorat 37 °C for 1 h. The absorbance of the samples in the plate was measured using a VersaMax microplate reader (Molecular Devices, San Jose, CA, USA).

### 2.4. Nitric Oxide Assay

The ImKCs were seeded in a 96-well plate and incubated overnight in a CO_2_ incubator at 37 °C. The cells were pretreated with CDG (5, 10, 20, and 40 μM) for 2 h. Further, they were treated with LPS (1 μg/mL) for 24 h in a CO_2_ incubator. The supernatants (100 μL) were then transferred into a new plate, followed by the addition of 100 μL of the Griess reagent (1% sulfanilamide, 0.1% N-1-naphthylenediamine dihydrochloride, and 2.5% phosphoric acid). The absorbance of the samples in the plate was measured using a microplate reader at 540 nm.

### 2.5. Enzyme-Linked Immunosorbent Assay (ELISA)

ELISA was used to measure the levels of pro-inflammatory cytokines produced by the cells. The ImKCs were cultured in a 96-well plate and treated with CDG (5, 10, 20, and 40 μM) for 2 h. Then, the cells were treated with LPS (1 μg/mL) and incubated in a CO_2_ incubator at 37 °C for 24 h. After incubation, the supernatants (100 μL) were transferred to another 96-well plate, and the level of each cytokine was measured using anti-interleukin-6 (IL-6), anti-interleukin-1β (IL-1β), and anti-tumour necrosis factor-α (TNF-α) antibodies (BD Pharmingen, San Diego, CA, USA). Purified antibodies were added to an ELISA plate, followed by incubation overnight in a refrigerator at 4 °C. Next, the antibodies were washed with the wash buffer (phosphate-buffered saline (PBS) with 0.05% Tween 20) and incubated with blocking solution (10% FBS in PBS) for 1 h at room temperature. After 1 h, the plate was rewashed with the wash buffer; then, the supernatant and standard solution were added, allowed to react for 1 h, and then washed with the wash buffer. After washing, the detection antibodies were added; the reaction was allowed to occur for 1 h. Next, streptavidin-conjugated alkaline phosphatase (AKP; BD Pharmingen) was added after washing; the reaction was allowed to occur for 30 min. After washing with a wash buffer, 1 tablet of 4-nitrophenyl phosphate (Sigma-Aldrich) was added to the substrate buffer (10% diethanolamine, 0.1% MgCl_2_·6H_2_O, 0.2% NaN_3_, pH 9.8); this solution was then added to each well, and when a color change was seen in the reference well, stop buffer (1 N NaOH) was added to stop the reaction. Finally, the absorbance of the samples was measured at 405 nm using a microplate reader.

### 2.6. RNA Extraction and cDNA Synthesis

The ImKCs were seeded in a 12-well plate and incubated overnight in a CO_2_ incubator. The cells were pre-treated with CDG (5, 10, 20, and 40 μM) for 2 h and were then activated by LPS (1 μg/mL) treatment for 3 h. The RNA was extracted from the ImKCs using a Trizol kit (Bio Science Technology, Daegu, Korea). The cDNA synthesis (conversion of RNA into cDNA by reverse-transcription) was performed using the iScript™ cDNA Synthesis Kit (Bio-Rad Laboratories, Hercules, CA, USA).

### 2.7. Quantitative Polymerase Chain Reaction (qPCR)

The cDNA obtained through reverse transcription was mixed with TOPreal™ qPCR 2× PreMIX (SYBR-Green, Enzynomics, Daejeon, Korea), according to the manual provided by the manufacturer. The PCR analysis was performed following the manufacturer’s protocol using CFX Connect Real-Time PCR Detection System (Bio-Rad Laboratories; pre-denaturation at 95 °C for 15 min, 38 cycles of denaturation at 95 °C for 10 s, annealing at 60 °C for 15 s, and extension at 72 °C for 30 s). The primers used for quantifying the target genes, including those encoding iNOS, IL-6, IL-1β, TNF-α, which are inflammatory mediators; *β-actin*; and glyceraldehyde 3-phosphate dehydrogenase (GAPDH) (Bionics, Seoul, Korea), which was used as the housekeeping gene, are shown in Table S1.

### 2.8. Western Blotting

The cells were cultured in a 12-well plate and incubated overnight in an incubator at 37 °C and treated with CDG for 2 h. After 2 h, cells were stimulated with LPS treatment for 3 min [phospho (p)-inhibitor of κB (IκB)α and IκBα] or 24 h (iNOS). The cells were washed with cold PBS and lysed in RIPA buffer (10 mM NaF, 100× protease inhibitor cocktail, 1 mM Na_3_VO_4_); the cell lysates were then transferred into microcentrifuge tubes. The cell pellets and the supernatants were separated by centrifugation, and the supernatants were used in the subsequent experiments. The prepared protein samples’ concentrations were measured at 595 nm using Bio-Rad Protein Assay Kit (Bio-Rad Laboratories). Bovine serum albumin (BSA) was used for the construction of the standard curve. The lysates were boiled in sample buffer at 100 °C for 5 min after mixing. The prepared samples were subjected to sodium dodecyl sulfate-polyacrylamide gel electrophoresis and transferred onto nitrocellulose membranes (XOGENE, Englewood, NJ, USA). Non-fat dry milk (5%, LPS Solution, Daejeon, Korea) in Tris-buffered saline-Tween 20 (TBS-T) was used as the blocking agent for each membrane. Then the membranes were incubated with the primary antibodies overnight at 4 °C in a refrigerator. Further, each membrane was incubated for 1 h with the secondary antibodies (Cell Signaling Technology, Danvers, MA, USA). The intensities of the protein bands on the membranes were visualized and analyzed using the AI680 system (GE Healthcare, Chicago, IL, USA), along with an electrochemiluminescence (ECL) solution.

### 2.9. Immunofluorescence Technique

Immunofluorescence was used to confirm the translocation of NF-κB p65, which leads to activation of the NF-κB pathway, and AT-rich interaction domain 5A (Arid5a), which post-transcriptionally regulates the stability of IL-6 mRNA. The ImKCs were cultured in a 12-well plate with a coverslip attached and pre-treated with CDG for 2 h. Next, cells were stimulated with LPS treatment for 2 h (p65) or 3 h (Arid5a). After LPS treatment, all the supernatants were removed. The cells were washed with PBS and fixed using 4% paraformaldehyde solution in PBS (Tech & Innovation, Chuncheon, Korea) on the coverslip at room temperature for 15 min. After fixation, the cells were washed with PBS and were permeabilized using 0.3% Triton X-100 (Sigma-Aldrich) on the coverslip for 10 min at room temperature and then washed with PBS. After permeabilization, blocking was performed at room temperature for 1 h using 1% BSA in PBS; then, the cells were washed with PBS. Thereafter, the samples were treated with dilute solutions of the primary antibodies at room temperature for 1 h, washed with PBS, and then reacted with diluted solutions of the secondary antibodies in the dark for 40 min. Finally, the samples were washed with PBS. The coverslips were fixed onto slide glasses using a mounting solution, and fluorescent images were captured using a Lionheart FX microscope (Biotek, Winooski, VT, USA).

### 2.10. Statistical Analysis

The data are expressed as the mean ± standard deviation. All experiments were performed in triplicate, with each being repeated three times. One-way ANOVA and Dunnett’s multiple comparison test were used to test the statistical significance of the results. The experimental results were considered statistically significant at *p* < 0.05.

## 3. Results and Discussions

### 3.1. Isolation and Identification of Catechin-7,4′-O-Digallate from W. uniflora

**Figure 1:**
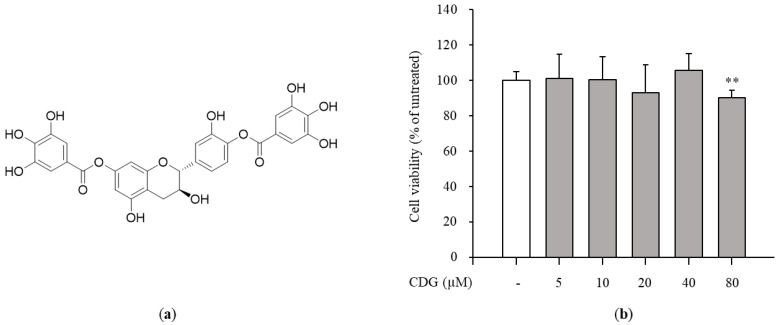
Effect of CDG on cell viability. (**a**) Chemical structure of CDG. (**b**) The immortalized mouse Kupffer cells (ImKCs)cells were treated with CDG (5, 10, 20, 40, and 80 μM) for 24 h. Cell viability rates were compared with the CDG-untreated control group. The cell viability rates are shown via a bar graph. Data are represented as the mean ± standard deviation. ** *p* < 0.01 relative to the CDG-untreated control group. CDG, catechin-7,4′-*O*-digallate; ImKC, immortalized mouse Kupffer cells.

The crude extract of *W. uniflora* leaves was partitioned using the solvents EtOAc and *n*-BuOH for fractionation to obtain EtOAc- and *n*-BuOH-soluble fractions. Based on the LC/MS analysis of the two fractions, further phytochemical investigation of the EtOAc-soluble fraction was carried out, by successive column chromatography including silica gel, RP-C18 silica, and Sephadex LH-20, as well as preparative and semi-preparative HPLC; this procedure resulted in the isolation of a polyphenol. Its structural elucidation was performed; the compound was confirmed to be CDG (Figure 1a) by comparing the NMR spectral data and optical rotation values with those reported earlier [22] and by LC/MS analysis.

Polyphenols have been shown to have a wide range of biological activities such as antioxidant and anticancer effects, as well as anti-inflammatory effects [23,24,25,26,27]. A polyphenol extracted from green tea, known as epigallocatechin gallate, is a famous anti-inflammatory and antioxidant compound [28]. Moreover, quercetin, which is abundant in fruits, vegetables, and leaves, has already been used to treat inflammation. Considering the anti-inflammatory potential of polyphenols, the discovery of a new polyphenol and the measurement of its anti-inflammatory effects is promising for the development of new anti-inflammatory drugs. Therefore, we evaluated the anti-inflammatory properties of a new polyphenol, CDG, and its regulatory mechanism of action in macrophages.

### 3.2. Inhibition of NO Production and iNOS Expression by CDG in LPS-Stimulated ImKCs

**Figure 2:**
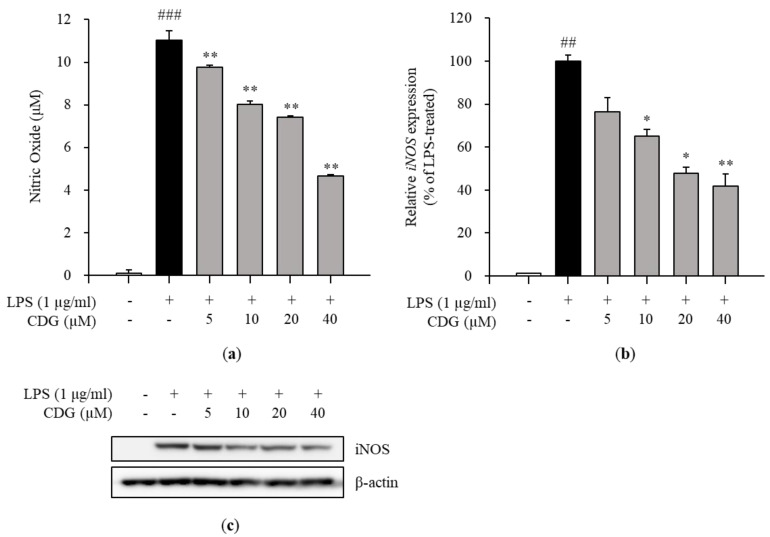
Effect of CDG on NO production and iNOS expression. The ImKC cells were treated with CDG (5, 10, 20, and 40 μM) for 2 h and stimulated by LPS (1 μg/mL) for the experiment. (**a**) After LPS treatment for 24 h, the NO production was measured and quantified using a standard curve for nitrite solution. The NO production level is shown via a bar graph. Data are represented as the mean ± standard deviation. (**b**) After LPS treatment for 3 h, the mRNA expression of iNOS was measured and compared with LPS-treated control group set at 100%. iNOS mRNA expression levels are shown with a bar graph. Data are represented as the mean ± standard deviation. (**c**) After LPS treatment for 24 h, the protein expression levels of iNOS were measured and compared with those in the LPS-treated control group. ^##^ *p* < 0.01 and ^###^ *p* < 0.001 relative to the untreated control group. * *p* < 0.05 and ** *p* < 0.01 relative to the LPS-treated and CDG-untreated group. CDG, catechin-7,4′-*O*-digallate; NO, nitric oxide; iNOS, inducible nitric oxide synthase; LPS, lipopolysaccharide.

The cytotoxicity test was performed to confirm the anti-inflammatory effects of CDG at non-cytotoxic concentrations. The ImKCs were treated with CDG for 2 h, followed by LPS treatment for 24 h. CDG was non-cytotoxic at concentrations up to 40 μM and cytotoxic at 80 μM. Hence, we used CDG at concentrations of up to 40 μM in this study (Figure 1b). NO is a signaling molecule that plays a key role in inflammatory responses. Overproduction of NO induces inflammatory reactions and tissue damage. ImKCs were treated with CDG for 2 h before stimulation with LPS treatment, resulting in reduced NO production, which occurred in a CDG dose-dependent manner (Figure 2a). In addition, we measured the iNOS mRNA and protein expression levels to confirm whether LPS-induced NO production is due to iNOS regulation. The mRNA and protein expression levels of iNOS were increased after LPS treatment and reduced after CDG treatment in a dose-dependent manner (Figure 2b,c). This result demonstrated that CDG treatment reduced NO production via downregulating iNOS expression.

### 3.3. Regulation of Pro-Inflammatory Cytokine Production by CDG in LPS-Stimulated ImKCs

**Figure 3:**
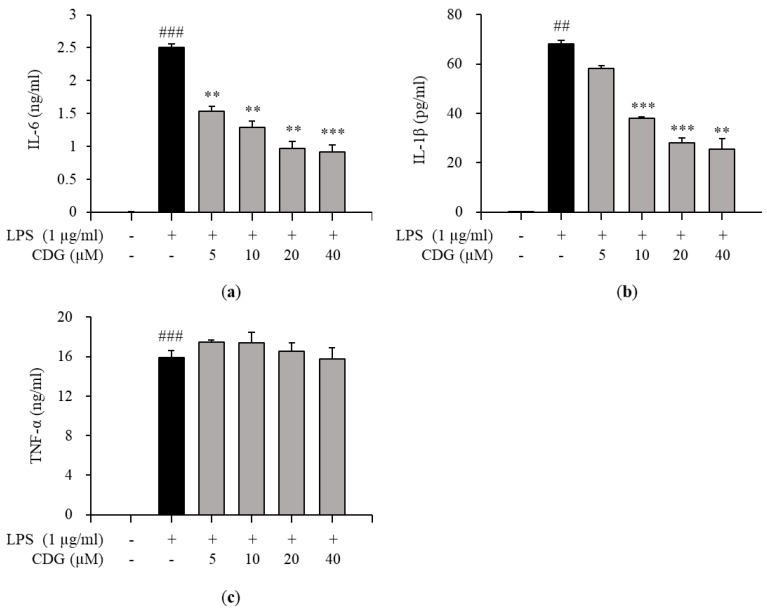
Effect of CDG on pro-inflammatory cytokine production. The ImKC cells were treated with CDG (5, 10, 20, and 40 μM) for 2 h and stimulated by LPS (1 μg/mL) for 24 h. After LPS treatment, the pro-inflammatory cytokine such as (**a**) IL-6, (**b**) IL-1β, and (**c**) TNF-α production were measured and quantified using a standard curve. The pro-inflammatory cytokine production level is shown with a bar graph. Data are presented as the mean ± standard deviation. ^##^ *p* < 0.01 and ^###^ *p* < 0.001 relative to the untreated control group. ** *p* < 0.01 and *** *p* < 0.001 relative to the LPS-treated and CDG-untreated group. CDG, catechin-7,4′-*O*-digallate; LPS, lipopolysaccharide; IL, interleukin; TNF, tumor necrosis factor.

**Figure 4:**
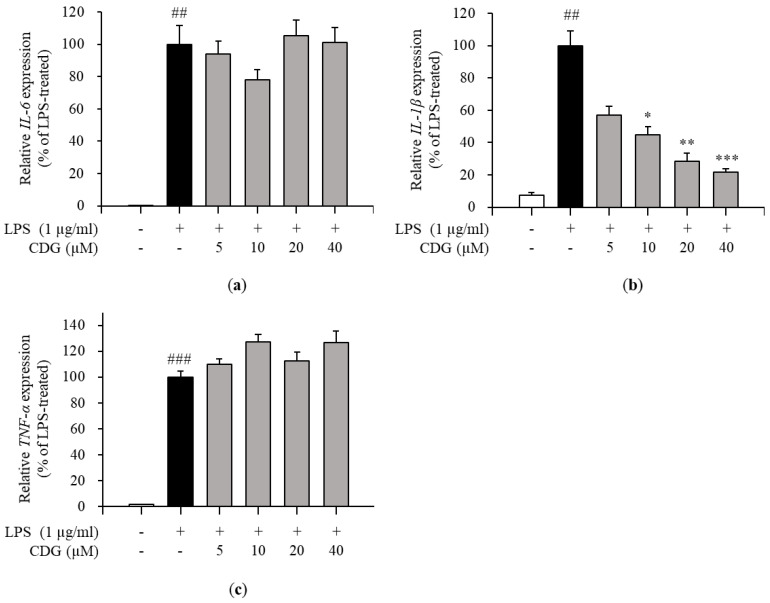
Effect of CDG on pro-inflammatory cytokine mRNA expression. The ImKC cells were treated with CDG (5, 10, 20, and 40 μM) for 2 h and stimulated by LPS (1 μg/mL) for 3 h. After LPS treatment, the mRNA expression of pro-inflammatory cytokines, such as (**a**) IL-6, (**b**) *IL-1β*, and (**c**) TNF-α, were compared with the LPS-treated group set at 100%. The mRNA levels of the pro-inflammatory cytokines are shown via a bar graph. Data are represented as the mean ± standard deviation. ^##^ *p* < 0.01 and ^###^ *p* < 0.001 relative to the untreated control group. * *p* < 0.05, ** *p* < 0.01, and *** *p* < 0.001 relative to the LPS-treated and CDG-untreated group. CDG, catechin-7,4′-*O*-digallate; LPS, lipopolysaccharide; IL, interleukin; TNF, tumor necrosis factor.

Inflammatory responses are promoted by inflammatory mediators, such as IL-6, IL-1β, TNF-α, and NO [29]. Therefore, the effects of CDG on the production of pro-inflammatory cytokines in LPS-treated ImKCs were investigated by ELISA. CDG treatment inhibited the production of IL-6 and IL-1β in a dose-dependent manner (Figure 3a,b), but not TNF-α (Figure 3c). Moreover, the mRNA expression levels of pro-inflammatory cytokines were measured by qPCR. CDG treatment reduced the expression of IL-6 and IL-1β, but not TNF-α, in a dose-dependent manner (Figure 4).

Via ELISA, we demonstrated that the levels of IL-6 and IL-1β production were reduced by CDG treatment. Next, via qPCR, it was confirmed that only the mRNA expression levels of *IL-1β* were affected by CDG treatment. In general, quantifying cytokine-encoding genes’ mRNA levels is the first step towards the production and regulation of cytokines. However, interestingly, we observed that the production of IL-6 was reduced despite the *IL-6* mRNA expression not being inhibited.

### 3.4. Inhibition of Phosphorylation and Degradation of IκBα by CDG in LPS-Stimulated ImKCs

**Figure 5:**
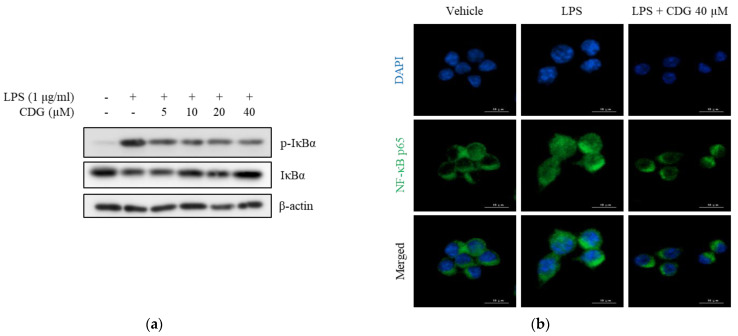
Effect of CDG on the NF-κB pathway. ImKC cells were treated with CDG (5, 10, 20, and 40 μM) for 2 h and stimulated by LPS (1 μg/mL) for the experiment. (**a**) Cell lysates were used for the analysis of the phosphorylation and degradation of IκBα by Western blotting. β-actin was used as the loading control. (**b**) Immunofluorescence staining for NF-κB p65 (green) and compared with DAPI (blue). Scale bars: 10 μm. CDG, catechin-7,4′-*O*-digallate; NF-κB, nuclear factor kappa-light-chain-enhancer of activated B cells; LPS, lipopolysaccharide; DAPI, 4′,6-diamidino-2-phenylindole. Scale bar = 10 μM.

Major signaling pathways for LPS-induced activation of inflammatory response in macrophages are mitogen-activated protein kinases (MAPKs) and NF-κB [30,31]. To elucidate the regulatory mechanism of action of CDG-mediated anti-inflammatory responses, the alleviation of MAPK phosphorylation by CDG was investigated in LPS-stimulated macrophages; however, CDG did not change phosphorylation status of MAPKs (Figure S1). NF-κB, another major inflammatory signaling pathway, is strictly regulated by IκBα, an NF-κB inhibitor. Upon LPS stimulation, IκBα is phosphorylated and then degraded by ubiquitination. NF-κB p50/p65 separate from IκBα and translocate into the nucleus, thereby activating the NF-κB pathway. Activated NF-κB induces the production of inflammatory mediators, such as NO and pro-inflammatory cytokines [32,33]. We measured the levels of IκBα phosphorylation and NF-κB p65 translocation to confirm NF-κB activation. The levels of p-IκBα were increased after LPS treatment and reduced after CDG treatment in a dose-dependent manner. In addition, LPS-induced degradation of IκBα was reduced by CDG treatment in a dose-dependent manner (Figure 5a).

NF-κB p65 translocation was confirmed using immunofluorescence assay. After LPS treatment, NF-κB p65 translocated from the cytoplasm to the nucleus, indicating NF-κB activation. However, CDG treatment (40 μM) blocked the nuclear translocation of NF-κB p65 (Figure 5b). Collectively, CDG inhibited IκBα degradation and phosphorylation as well as the activation of NF-κB by blocking the nuclear translocation of NF-κB p65, eventually leading to reduced NO production.

Based on the experimental data, CDG appears to selectively regulate iNOS and IL-1β production based on the selective regulation of the NF-κB pathway. Several studies have shown that natural compounds cannot inhibit the production of multiple pro-inflammatory cytokines simultaneously. Depending on the inhibitory effects of compounds on only certain signaling pathways, the production of cytokines may be differentially regulated by various compounds [34,35]. In particular, p38 is the main regulator of TNF-α mRNA expression [36], and there is a study showing that the downregulation of the components of only the NF-κB pathway cannot completely inhibit TNF-α production [37]. Furthermore, extracellular signal-regulated kinase (ERK) is not essential for the production of IL-1β. Based on these studies, we suggest that the inhibition of iNOS and IL-1β transcriptional activation by CDG treatment might be due to its selective regulatory effect on NF-κB.

### 3.5. Inhibition of Arid5a Activation by CDG in LPS-Stimulated ImKCs

**Figure 6:**
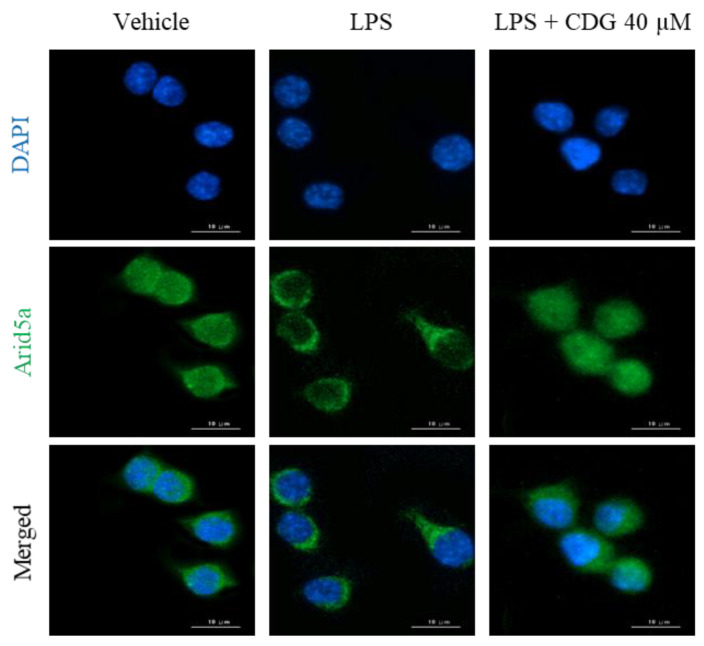
Effect of CDG on Arid5a activation. The ImKC cells were treated with CDG (5, 10, 20, and 40 μM) for 2 h and stimulated by LPS (1 μg/mL) for 3 h. Immunofluorescence staining for Arid5a (green) and compared with DAPI (blue). Scale bars: 10 μm. CDG, catechin-7,4′-*O*-digallate; ImKC, immortalized mouse Kupffer cells; LPS, lipopolysaccharide; Arid5a, AT-Rich interaction domain 5a; DAPI, 4′,6-diamidino-2-phenylindole. Scale bar = 10 μM.

In general, cytokine production is tightly regulated by the transcriptional activation of cytokine-encoding genes. In the present study, though IL-6 secretion was regulated by CDG, its mRNA expression remained unaffected, as revealed by the ELISA and qPCR results (Figure 3 and Figure 4). Based on these results, we speculated that CDG treatment does not affect *IL-6* mRNA expression but is involved in the regulation of RNA-binding proteins (RBPs), such as tristetraprolin (TTP), AT-rich interaction domain 5A (Arid5a), and regnase-1, which affect its post-transcriptional stability, thereby regulating its secretion [38]. Arid5a is an RBP that specifically increases the post-transcriptional stability of IL-6 mRNA, while TTP and regnase-1 affect the mRNA stability of other cytokines in addition to IL-6 [39,40]. Furthermore, it has been reported that Arid5a and regnase-1 act competitively, but Arid5a dominates regnase-1 [41,42,43]. Under normal conditions, Arid5a exists in the nucleus. It is activated by LPS stimulation and is then translocated to the cytoplasm, where it interacts with the three prime untranslated regions (3′-UTR) of IL-6 mRNA, enhancing the stability of IL-6 mRNA [44,45]. We performed immunofluorescence analysis to demonstrate the effect of CDG on the localization of Arid5a in LPS-stimulated macrophages. Following LPS stimulation, Arid5a was found to have translocated from the nucleus to the cytoplasm. CDG treatment (40 μM) inhibited the LPS-induced translocation of Arid5a to the cytoplasm (Figure 6). This result indicates that CDG treatment reduced the stability of IL-6 mRNA and thus its secretion by inhibiting the cytoplasmic localization of Arid5a in LPS-stimulated macrophages.

## 4. Conclusions

This study aimed to investigate the anti-inflammatory effects of CDG. NO production was induced by LPS treatment and inhibited by CDG treatment. Additionally, the production of the pro-inflammatory cytokines IL-6 and IL-1β, but not TNF-α, was reduced by CDG treatment. The levels of IκBα phosphorylation, degradation, and hence the nuclear translocation of NF-κB p65 were reduced by CDG treatment. The mechanism underlying the regulation of IL-6 production by CDG involved reducing the stability of IL-6 mRNA by preventing the cytoplasmic translocation of Arid5a. These results suggested that the mechanism underlying the anti-inflammatory effects of CDG was mediated through downregulation of the NF-κB pathway and inhibition of cytoplasmic localization of Arid5a. In conclusion, CDG has the potential to be developed as a drug for treating inflammatory diseases.

## Data Availability

The data presented in this study are available in the article or Supplementary Material.

## Supplementary Materials

The following are available online at https://www.mdpi.com/1999-4923/13/3/408/s1, General experimental procedure, plant material, Figure S1: Effect of CDG on the MAPK pathway, Figure S2: ^1^H NMR spectrum of catechin-7,4′-*O*-digallate (CDG) in CD_3_OD, Figure S3: LC/MS analysis of CDG, Table S1: List of the primer sequences for qPCR.
