# Supplementary material for: Anti-Inflammatory Effects of a Polyphenol, Catechin-7,4′-O-Digallate, from Woodfordia uniflora by Regulating NF-κB Signaling Pathway in Mouse Macrophages
Source: Pharmaceutics. 2021 Mar 19;13(3):408. doi: 10.3390/pharmaceutics13030408 (PMC8003360; doi:10.3390/pharmaceutics13030408)
Supplement: Supplementary file 1 [file pharmaceutics-13-00408-s001.pdf]

pharmaceutics

# Supplementary Materials:Anti-inflammatory Effects of a Polyphenol, Catechin-7,4′-O-digallate, from Woodfordia uniflora by Regulating NF-κB Signaling Pathway in Mouse Macrophages

**Seoyoung Lee, Eui Jin Kim, Jae Sik Yu, Ji Bin Seo, Jae Sung Lim, Jeong Uk Choi, Chang-Min Lee, Luay Rashan, Ki Hyun Kim*, and Young-Chang Cho***

## General experimental procedure

Optical rotation was measured using Jasco P-2000 polarimeter (Jasco, Easton, MD, USA). Ultraviolet (UV) spectra were obtained using Agilent 8453 UV-visible spectrophotometer (Agilent Technologies, Santa Clara, CA, USA). Nuclear magnetic resonance (NMR) spectra were obtained using Bruker AVANCE III 850 NMR spectrometer with 5 mm TCI CryoProbe, operating at 850 MHz ($^1$H) and 212.5 MHz ($^{13}$C), with the chemical shift values being represented as parts per million (ppm). Preparative high-performance liquid chromatography (HPLC) was performed using a Waters 1525 Binary HPLC pump with Waters 996 Photodiode Array Detector (Waters Corporation, Milford, CT, USA). Semi-preparative HPLC was performed using the Shimadzu Prominence HPLC System with SPD-20A/20AV Series Prominence HPLC UV-Vis Detectors (Shimadzu, Tokyo, Japan). LC/MS analysis was performed on an Agilent 1200 Series HPLC system, equipped with a diode array detector and 6130 Series ESI mass spectrometer. An analytical Kinetex $C_{18}$ 100 Å column (100 × 2.1 mm, 5 μm; flow rate: 0.3 ml/min; Phenomenex) was used. Furthermore, the Dianion HP-20 system (Mitsubishi Chemical, Tokyo, Japan) along with silica gel 60 (Merck, 230–400 mesh) and RP-$C_{18}$ silica gel (Merck, 40–63 μm) were used for column chromatography, Merck pre-coated Silica gel F254 plates and RP-18 F254s plates (Merck, Darmstadt, Germany) were used for thin-layer chromatography (TLC). The plates were visualized under UV light or by heating after spraying with anisaldehyde-sulfuric acid until the development of spots.

## Plant material

Leaves of *W. uniflora* were collected from the valleys of Ain Jarziz, which is approximately 17–18 km east of Salalah, Dhofar (Oman), during June–August 2017. The leaves were dried at room temperature, macerated into a fine powder, and stored at room temperature. The plant was classified by Alan Radcliffe-Smith (Royal Botanic Gardens Kew, England) [1]. A voucher specimen of the material (W-2017) was deposited at the Biology Department, Sultan Qaboos University, Sultanate of Oman.

**Publisher's Note:** MDPI stays neutral with regard to jurisdictional claims in published maps and institutional affiliations.

**Copyright:** © 2021 by the authors. Submitted for possible open access publication under the terms and conditions of the Creative Commons Attribution (CC BY) license (http://creativecommons.org/licenses/by/4.0/).

## Reference

1.Miller, A.G.; Morris, M.; Stuart-Smith, S. *Plants of Dhofar, the southern region of Oman: traditional, economic, and medicinal uses*; Office of the Advisor for Conservation of the Environment, Diwan of Royal Court., Sultanate of Oman: 1988.

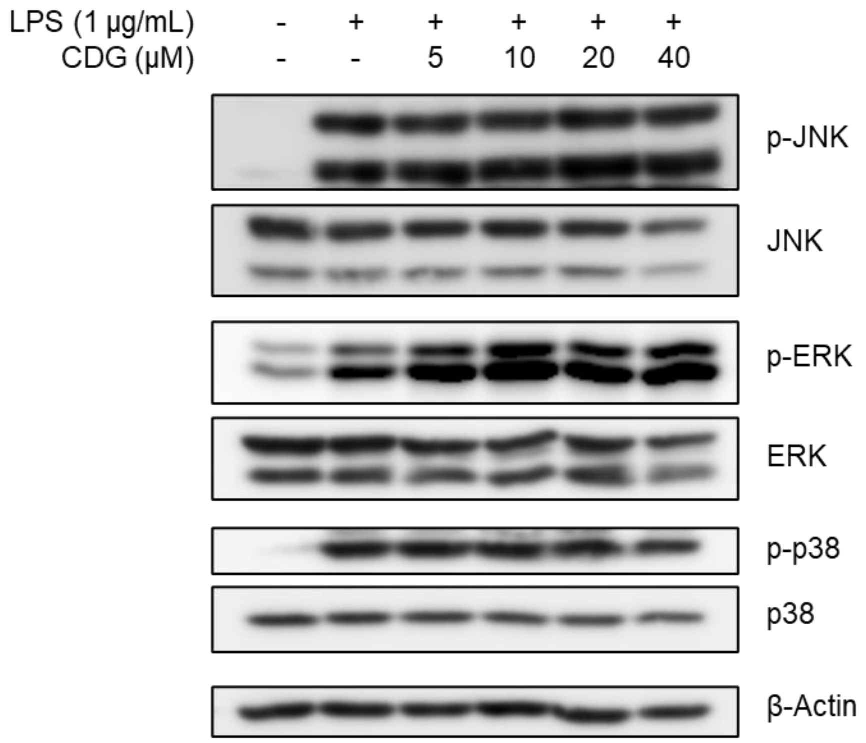


**Figure 1.** Effect of CDG on the MAPK pathway. The ImKC cells were treated with CDG (5, 10, 20, and 40 μM) for 2 h and stimulated by LPS (1 μg/mL) for 15 min. The cell lysates were used for the analysis of the phosphorylation of MAPK (ERK, JNK, and p38) by western blotting. β-actin was used as the loading control. CDG, catechin-7,4′-O-digallate; MAPK, mitogen-activated protein kinase; LPS, lipopolysaccharide. ERK, extracellular signal-regulated kinase; JNK, c-Jun N-terminal kinase.

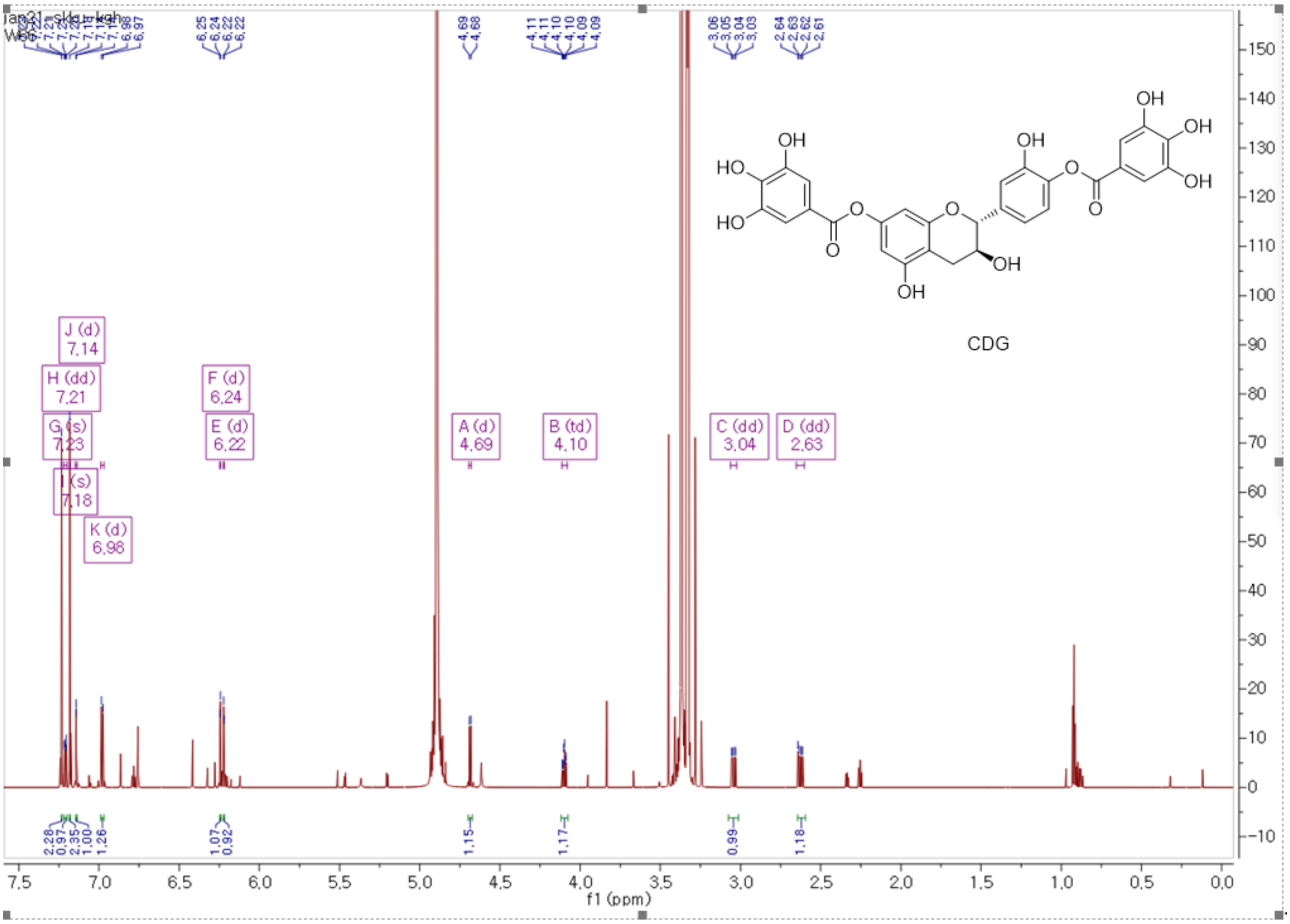


**Figure S2.** $^{1}$H NMR spectrum of catechin-7,4′-*O*-digallate (CDG) in $CD_3OD$.

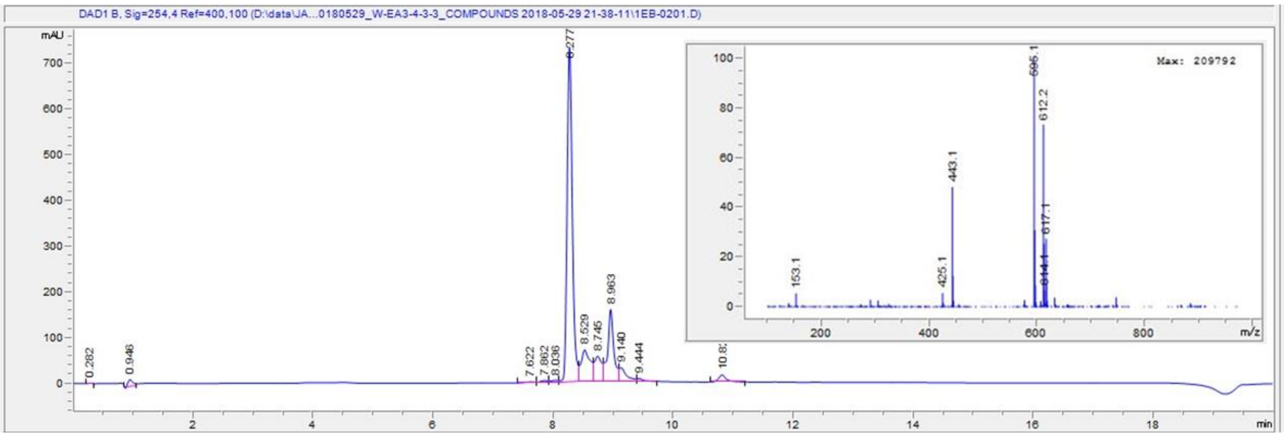


**Figure S3.** LC/MS analysis of CDG [ESIMS (positive-ion mode) *m*/*z*: 595.1 [M + H]$^+$ and *m*/*z*: 617.1 [M + Na]$^+$. Detection wavelength was set at 254 nm; LC/MS analysis was performed on an Agilent 1200 Series HPLC system equipped with a diode array detector and 6130 Series ESI mass spectrometer using an analytical Kinetex C18 100 Å column (100 × 2.1 mm, 5 µm; flow rate: 0.3 mL/min; Phenomenex). The mobile phase was composed of 0.1% (w/v) formic acid in water (solvent A) and methanol (solvent B) in the following gradients; 0 min (A:B 90:10), 10 min (A:B 10:90), 11 min (A:B 0:100), 15 min (A:B 0:100), 16 min (A:B 90:10), and 20 min (A:B 90:10).].

**Table S1.** List of the primer sequences for qPCR. Primer sequences for inflammatory mediators and housekeeping genes. qPCR, quantitative PCR; iNOS, inducible nitric oxide synthase; IL, interleukin; TNF, tumor necrosis factor; GAPDH, glyceraldehyde-3-phosphate dehydrogenase.

| **Target gene** | | **Primer Sequence** |
|---|---|---|
| iNOS | Sense | 5′-TGG CCA AGC TGA ACT-3′ |
| | Anti-Sense | 5′-TCA TGA TAA CGT TTC TGG CTC TT-3′ |
| IL-6 | Sense | 5′-TCT AAT TCA TAT CTT CAA CCA AGA GG -3′ |
| | Anti-Sense | 5′-TGG TCC TTA GCC ACT CCT TC-3′ |
| IL-1β | Sense | 5′-TTG ACG GAC CCC AAA AGA-3′ |
| | Anti-Sense | 5′-GAT GTG CTG CTG CGA GAT T-3′ |
| TNF-α | Sense | 5′-CTG TAG CCC ACG TCG TAG C-3′ |
| | Anti-Sense | 5′-TTG AGA TCC ATG CCG TTG-3′ |
| GAPDH | Sense | 5′-AAC TTT GGC ATT GTG GG-3′ |
| | Anti-Sense | 5′-ACA CAT TGG GGG TAG GAA CA-3′ |
| β-actin | Sense | 5′-TGT TAC CAA CTG GGA CGA CA-3′ |
| | Anti-Sense | 5′-GGG GTG TTG AAG GTC TCA AA-3′ |
